# Social ecological factors affecting substance abuse in Ghana (West Africa) using photovoice

**DOI:** 10.11604/pamj.2019.34.214.12851

**Published:** 2019-12-30

**Authors:** Ahmed Kabore, Evans Afriyie-Gyawu, James Awuah, Andrew Hansen, Ashley Walker, Melissa Hester, Moussa Aziz Wonadé Sié, Jarrett Johnson, Nicolas Meda

**Affiliations:** 1 Jiann-Ping Hsu College of Public Health, Statesboro, Georgia Southern University, Georgia, United States; 2 Centre Muraz, Department of Public Health, Bobo-Dioulasso, Burkina Faso; 3 Joseph Ki-Zerbo University, Department of Public Health, Ouagadougou, Burkina Faso; 4 University of South Dakota, Sanford School of Health Sciences, South Dakota, United States

**Keywords:** Substance abuse, illicit drugs, drug addiction, mental health, risk factors, protective factors, West Africa

## Abstract

**Introduction:**

Substance abuse is an important public health issue affecting West Africa; however, there is currently a dearth of literature on the actions needed to address it. The aim of this study was to assess the risks and protective factors of substance abuse in Ghana, West Africa, using the photovoice method.

**Methods:**

This study recruited and trained 10 participants in recovery from substance abuse and undergoing treatment in the greater Accra region of Ghana on the photovoice methodology. Each participant received a disposable camera to take pictures that represented the risk and protective factors pertinent to substance abuse in their communities. They were also given the opportunity to provide narratives of the pictures using pre-identified themes and the different levels of the social-ecological model and participatory action research (PAR).

**Results:**

Participants identified at the individual level: ignorance; interpersonal level: family and peer pressure; organizational level: lack of regulation; community level: media, availability of drugs, cost of drugs, urbanization, slum communities and cultural factors; and policy level: lack of regulations and their enforcement. Education and beliefs were cited at the individual level; family at the interpersonal level; religion at the organizational level; organizing youth, media and narcotics anonymous at the community level; and nothing at the policy level.

**Conclusion:**

This is an exploratory study that will add to the limited body of knowledge in the scientific literature with respect to substance abuse in the country and also help develop interventions to address the respective needs of several communities in Ghana.

## Introduction

Drug abuse continues to be a controversial issue around the globe. It leads to enormous loss in life, and its effects spill over into the general society as well, thereby hindering the effective functionality and growth of populations [1]. In developing countries, for instance, the harm is magnified by the poor economic and living conditions. Government spending on substance abuse treatment is seen as an extra financial burden on an already strained budget. Furthermore, drug addiction can threaten civic safety, given that people under the influence of drugs may have a distorted view of the world around them and are prone to participating in improper behaviors such as armed robbery, traffic violations, and domestic violence [2]. Such persons can then become a danger to their family, the general public, and the environment [3]. Substance abuse also increases the risk of spreading infectious diseases such as HIV/AIDS, hepatitis B and C and tuberculosis. Drug abusers tend to share infected needles and cannot make appropriate decisions while under the influence of controlled substances, which can lead to the spread of the above-mentioned infections. Additionally, being under the influence of illicit drugs can also increase the incidence of unprotected sex [4]. Studies on substance abuse and potential intervention strategies in Africa are extremely limited in the scientific literature, highlighting the need for more research on this subject. Thus, the primary objective of this study was to assess the risk and protective factors pertinent to substance abuse in Ghana, West Africa, using the photovoice methodology.

## Methods

Photovoice is a process whereby cameras are given to a group of people to aid them in identifying the factors that harm or protect them or their communities [5]. This study involved 10 Ghanaian participants living in the Greater Accra region who were asked to identify the risk and protective factors associated with substance abuse in their communities. The study population comprised 9 men and 1 woman, and the average age of the participants was 42 years old. The study was guided by the social-ecological model and PAR theory. Combining PAR theory with the photovoice method was a highly beneficial method for engaging the Ghanaians to identify the problem in their community and reflect on the issue of substance abuse to propose an action plan. The study followed the nine steps of the photovoice methodology proposed by Wang and Burris as elaborated below [5].

**Step 1: select and recruit a target audience of policymakers or community leaders:** the study was conducted with the help of community leaders and the Korle-Bu Teaching Hospital´s drug addiction unit.

**Step 2: recruit a group of photovoice participants:** a purposeful sampling technique was used for this exploratory study. To ensure easy, in-depth discussion of the photos, 10 participants were invited to take part, as recommended for the photovoice methodology [6]. Overall, 12 participants were recruited based on their availability and the distance between their localities and the study site in Accra. The team met the participants to explain the purpose of the project and the role they would play if they decided to participate. Of the 12 participants who were recruited, 10 completed the project.

**Step 3: introduce the photovoice methodology to participants and facilitate a group discussion about cameras, power and ethics:** participants received 4hrs of training on photovoice. The first part of the training consisted of explaining to the participants why they were selected for the project, the purpose of the research project, and the risks and benefits involved as well as their rights as participants. Second, the team explained to the participants what photovoice is, why pictures are important, and what procedures are involved in the photovoice methodology. The participants were also advised on the ethical issues and potential risks, as well as how they could minimize them [7].

**Step 4: obtain informed consent:** written informed consent principles were read and explained to the participants, including the purpose of the project procedures and their significance. The consent document also informed participants of the potential risks of the project and made it clear that participation was voluntary. It stated that participants had the right to withdraw at any time for any reason [6].

**Step 5: pose initial theme(s) for taking pictures:** the participants received a specific theme that covered risk factors such as poverty, peer pressure, the media, cultural factors, ignorance and corruption, as well as a theme related to protective factors against substance abuse such as religion, family, education, and beliefs. The following questions were discussed with the aim of helping the participants understand the importance of the pictures and the potential benefits that could result and influence relevant policymaking based on the evidence presented to them: 1. What are the perceived sociological, economic, political, and cultural (ecological) risk factors for substance abuse in Ghana, according to patients in recovery? 2. What are the perceived sociological, economic, political, and cultural (ecological) protective factors against substance abuse in Ghana, according to patients in recovery?

**Step 6: distribute cameras to participants and review how to use them:** a disposable camera with an identification number was provided to each participant. The participants were also given the opportunity to try a demo camera provided during the training. They received a hands-on demonstration on how to use the camera for the project (i.e. how to use the flash, how to choose the proper distance from the target, and how to hold the camera when taking pictures [7]).

**Step 7: provide time for participants to take pictures:** the participants were given 1 week to take the pictures. After they returned the cameras, the research team developed and printed all the photos.

**Step 8: meet to discuss photographs and identify themes:** for logistical reasons, follow-up interviews were subsequently conducted instead of the group discussions frequently adopted in photovoice procedures. The first step was to select the photographs, contextualize them, and engage in pertinent storytelling [7]. The participants were asked the following questions: “What did you see here? What was really happening here? How does this relate to your life? Why is this situation a concern, or why does it exist? What can we do about it?” [7].

**Step 9: plan with participants a format to share photographs and stories with policymakers or community leaders:** the results of the study were made available in Accra, Ghana, during a community forum with the participation of political and religious leaders. The predominant topics included the stigmatization of drug users and ignorance (among individuals who do not use drugs) of the drug abuse situation in the country. Some religious leaders were surprised to see the pictures delineating the problems in their communities. After the forum, the religious and political leaders desired to learn more about substance abuse to develop data-driven strategies to address the issue and better provide aid to their communities.

## Results

The results were organized such that the first segment yielded tables that summarize the different quotes and themes, followed by pictures showing the risk and protective factors pertinent to substance abuse. The pictures were not sorted based on the different levels of the ecological model because some were used for multiple levels of the model (Table 1). Quotes based on the social-ecological model.

**Table 1:** Quotes based on the socio-ecological model

Levels of the socio-ecological model	Illustrative Quotes
**Individual level**	“People are starting drugs because they don’t know what they are doing. They are just ignorant.” (Participant 5)
	“In Ghana people are using drugs because they are poor and have nothing to lose. Idleness is the main source of the drug problems in this country.”(Participant 7)
**Interpersonal level**	“The kids learn from their parents, and what they see is what they would want to try.” (Participant 10)
	“Drugs are accessible to youths from family members, friends, or during underage partying.”(Participant 1)
**Organizational level**	“In school, children receive education about the dangers of using drugs so that by the time they reach 20 years of age, they already know what drugs can do to them. they are law stopping school children from smoking in the school setting, but the problem is the enforcement”(Participant 6)
	“The church in Ghana is playing an important role in youth education. People are taking seriously what a pastor says compare to a health educator.”(Participant 3)
**Community level**	“The majority of the people come to urban cities without prior financial preparation for the unknowns.”(Participant 3)
	“Alcohol advertisement outside the university campus. The central message of the advertisement is to influence people to buy alcohol. ” (Participant 4)
**Policy level**	“This is very common in our system. One problem is that the law enforcement tries to arrest the abuser rather than the ones selling, you know, the one bringing in the drugs to the system. ” (Participant 5)
	“I think that our police system doesn’t work. You know, this law enforcement, when they arrest you, you just need to give them something and it’s done.” (Participant 7)

**Figure 1:**
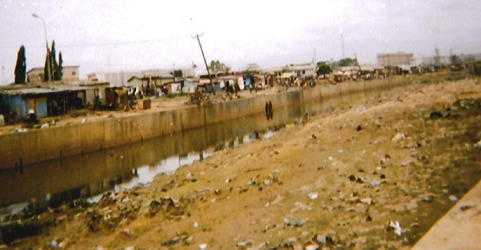
A photo of shanty towns in Accra illustrates the canal of drug addiction; the slums spread over 5km and the only business they do there is selling drugs

**Figure 2:**
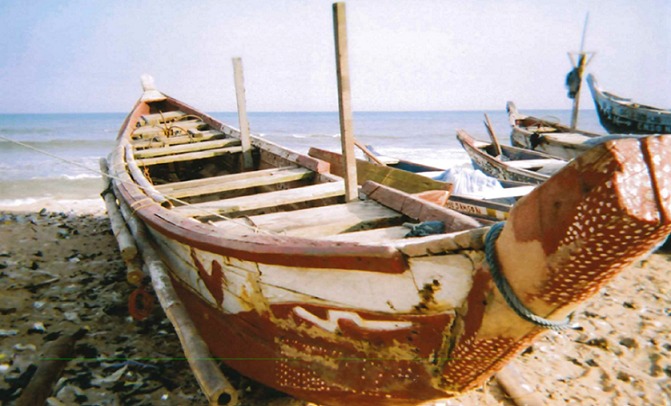
A canoe at the seashore: canoes are used to transport the drugs that come from South America from the high seas to the seashore, with canoes, it is easy to bring the drugs into Accra, Ghana

**Risk factors for substance abuse:** Table 2 indicates that, at the individual level, ignorance was often the reason that people use drugs. Family and peer pressure also helped explained why participants use drugs. At the organizational level, lack of regulation was given as a reason. Participants cited the media, the availability of drugs, the cost of drugs, urbanization, slum communities (an example of slums is shown in Figure 1 explaining how the problems of these communities feed the issue of substance abuse in Accra) and cultural norms as risk factors for substance abuse. At the policy level, lack of regulation/enforcement put participants at risk of using drugs (Table 2). Risk factors for substance abuse according to the recovery participants. Participant 6 indicated that drugs enter the country via the sea; they are transported on the high seas, offloaded into canoes and brought to the shore by local fishermen. An example of the canoes is shown in Figure 2.

**Table 2:** Risk factors to substance abuse according to participants in recovery

Individual	Interpersonal	Organizational	Community	Policy
Ignorance	Family	Lack of regulation	Media	Lack of regulations
	Peer – pressure		Availability of drugs	Lack of regulations enforcement
			Cost of drugs	
			Urbanization	
			Slum communities	
			Cultural factors	

**Figure 3:**
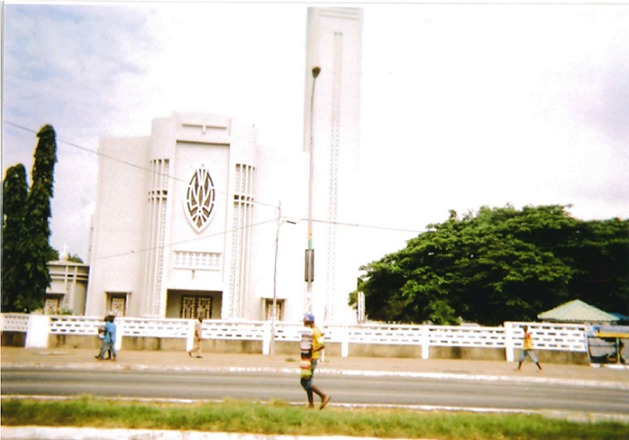
A photo of a church in Accra illustrates the role of religious groups in the fight against substance abuse in Ghana

**Figure 4:**
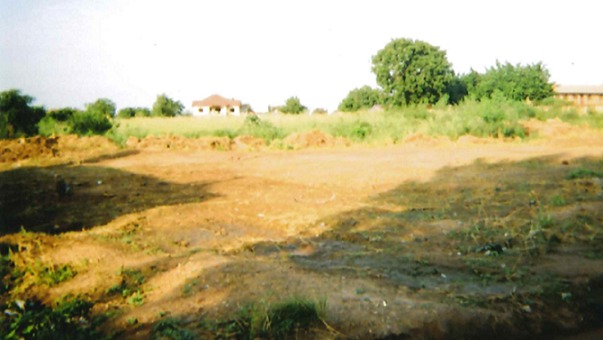
An open football field illustrates that youths in Ghana do not need much to practice a sport

**Protective factors against substance abuse:** Table 3 gave an overview of protective factors against substance abuse. Participants indicated that education and beliefs protect them from using drugs at the individual level. At the interpersonal and organizational levels, they cited family and religion as factors that stop participants from using drugs. Figure 3 provides an illustration of a church in Accra, highlighting the role of religious groups. Participant 5 indicated that the church and the Muslim community have been helping educate the people about the dangerous of substance abuse. At the community level, the participants declared that youth organizations, the media and Narcotics Anonymous protect them from drug use and abuse. Additionally, established intervention committees hold meetings and reach out to the elderly population in the communities regarding substance abuse, which is one of the few protective factors because much respect, culturally, is given to the elderly, who advise the youth about engaging in substance abuse (Table 3). Participant 4 discussed the role of sports and after-school programs as substance abuse prevention measures. Figure 4 illustrated a place where youths play football.

**Table 3:** Protective factors to substance abuse according to participants in recovery

Individual	Interpersonal	Organizational	Community	Policy
Education	Family	Religion	Organizing youth	
Beliefs			Media	
			Narcotic Anonymous	

## Discussion

The findings of this study indicated that multiple risk factors affect one's susceptibility to substance abuse. Several themes emerged in the study, but poverty was a frequent theme among the study participants, coupled with others such as corruption, urbanization and idleness. The primary factors preventing people from indulging in drug abuse were family values, religion, and education. The participants proposed some recommendations and a plan of action to develop substance abuse interventions. The main recommendations that emerged from the interviews were intensive sensitization to substance abuse and its effects, treatment and educational activities. Participants indicated that the primary solution to the problem is preventing the youth from exposure to drugs before they become interested in using them because the cost of treatment remains out of reach for the average Ghanaian. This study aimed at identifying the individual, interpersonal, organizational, community and policy factors affecting substance abuse in Ghana (West Africa). The photographs presented above and the information provided by the participants are indications that substance abuse continues to be an issue of concern in Ghana. Drugs affect not only those who abuse them but also society in general because drug use raises the crime rate and affects the youths´ education.

**Individual level:** the results of this study showed that poverty and idleness contribute to substance abuse in Ghana. When individuals do not have work, they are prone to indulging in social vices as a coping mechanism [8]. This has the potential to influence individuals' perceived self-worth, self-esteem, and purpose-driven direction [9]. Indulging in substance abuse appears to be a coping mechanism which individuals adopt when they have low expectations for their lives and little urge to pursue their own defined goals.

**Interpersonal level:** interpersonal interactions influence behaviors irrespective of age. Children and adolescents learn from their parents, and, as a society, we learn from one another. When children see a parent indulge in substance abuse, it increases their chances of trying those substances as well [10]. For instance, in Ghana, the drinking age is not well enforced for the underaged population; therefore, alcohol abuse may not be well regulated. In the absence of such enforcement of the law, children and adolescents may start abusing substances at an early age if they consider it an acceptable practice or social norm [11]. Participants indicated that without strong family values, many Ghanaian may be at risk of engaging in “bad” behaviors. Parents play an important role in influencing their children by serving as role models and by adopting responsible behavior with respect to drugs.

**Organizational level:** at the organizational level, religious groups play an important role in protecting individuals from drugs. Anti-substance activities are seen during Sunday school among Christians and frequent mosque visits among Muslims. One study looked at ways to prevent adolescents from engaging in substance abuse and proposed that changes be made in the church to address the issue of substance abuse, such as incorporating drug education in religious teachings [12]. In addition to the role that religious groups may play in preventing drug abuse, the school is an organization that may serve as a risk factor or a protective factor with respect to substance abuse.

**Community level:** at the community level, the participants reported the impact of slum communities on drug use or abuse. This can be explained by the fact that regulations in the slum communities are almost nonexistent. For example, with the lack of conventional roads or running water systems, it is difficult for the police or fire department to intervene in the case of an emergency. Additionally, drugs are readily accessible and available at low cost [13]. There is a need to organize the communities and create playgrounds for after-school programs. Participants indicated that the youth do not need excessive resources to change. A single ball for football (soccer) games and a field to engage in physical activities in a community could go a long way. A study that looked at youth sport programs suggested that sporting activities are ways to promote positive growth and development [14]. In Accra, when youth are involved in extracurricular activities, they do not normally think about engaging in drugs, which explains why the community would benefit from parks and recreational activities to occupy themselves [15].

**Policy level:** at the policy level, the participants did not identify a factor that protected them from drugs. Some studies indicate that policies should include information about drug education. This would be a good way to inform the youth and provide them with the knowledge they need to make more informed decisions. One report suggested mandatory drug testing as a way of discouraging students from drug usage, which would also allow schools to identify drug users and refer them to the right treatment support programs [16]. According to the study participants, corruption is a risk factor for drug use or abuse in Ghana. This could be explained by the fact that because of the level of poverty, it is easy to bribe the police to buy their silence [17]. Corruption influences or facilitates the entry of drugs into society. Collaboration between various forces of law and order could help reduce the vulnerability of law enforcement agents to accepting bribes. In Africa as a whole, the damage caused by substance abuse is not a priority because many countries are confronting other more pressing health and/or socioeconomic issues. As stated in an article focused on the drug trade in West Africa, many politicians want to tackle the issue of drugs but often face other more powerful individuals who do not view the drug issue as a priority [18]. Participants proposed an international collaboration to stop drugs from entering Ghana as a solution. Table 4 indicates the next steps that participants proposed to reduce the risk of substance abuse in their community.

**Table 4:** Action plan proposed by participants to reduce the risk of substance abuse in the community

Individual	Interpersonal	Organizational	Community	Policy
Educate people not to start using alcohol	Educate parents from using drugs in front of their children	Establish a structure to help underprivileged people	Breaking up slum communities	International collaboration to stop drug from entering Ghana
Educate individuals that the reality in the Western Work is not the same in Ghana			Help youth gain employment to avoid idleness	Better regulation of alcohol advertisement
Educate people about the risk of buying medicine of the street			Target individuals who sell drugs in the community	Enforcement of underage drinking law
			Fight corruptions	Enforcement laws preventing pharmacies from selling control substances without prescriptions

## Conclusion

This current study sought to explore the risk factors regarding substance abuse in Ghana, West Africa. Overall, the participants could identify why people use or abuse drugs from different perspectives, including ignorance at the individual level; family and peer pressure at the interpersonal level; lack of regulation at the organizational level; the availability of drugs, the cost of drugs, the media, urbanization, and slum communities at the community level; and lack of (or inadequate) enforcement and regulations at the policy level. The participants also identified potential protective factors against substance abuse in their communities. As described by participants, drug addiction is not viewed as a disease in Ghana, which makes it difficult to develop interventions. Addiction-related issues receive less attention from the population and at the policy level. Further studies are warranted to help educate the population about the dangers of drugs that are easily accessed on the streets of Ghana. Prevention remains the best option for addressing this issue of substance abuse because of the wide range of available treatment processes.

### What is known about this topic

- Literature has addressed the fact that substance abuse was a concern in West Africa;
- Literature has addressed the challenges of addressing the issue in the region.

### What this study adds

- Youth in slum communities sell drugs in order to survive which makes drugs such as marijuana and amphetamine available and at low cost;
- Education was highlighted as the most effective way to reach communities;
- The Church and Mosques were chosen as the most effective avenue to educate communities about the danger of substance abuse.

## Competing interests

The authors declare no competing interests.

## References

[1] Narcotics Control Board (2004). Report of the International Narcotics Control Board.

[2] Hohl BC, Wiley S, Wiebe DJ, Culyba AJ, Drake R, Branas CC (2017). Association of drug and alcohol use with adolescent firearm homicide at individual, family and neighborhood levels. JAMA Intern Med..

[3] Kellen A, Powers L, Birnbaum R (2017). Drug use, addiction and the criminal justice system. Responding Oppression Addict Can Soc Work Perspect..

[4] Chowa G, Masa R, Osei-Akoto I (2012). Youth and their health in Ghana. Youth Save Research Brief..

[5] Wang C, Burris MA (1997). Photovoice: Concept, methodology, and use for participatory needs assessment. Health Educ Behav..

[6] Wang CC, Cash JL, Powers LS (2000). Who knows the streets as well as the homeless? Promoting personal and community action through photovoice. Health Promot Pract..

[7] Wang CC (1999). Photovoice: a participatory action research strategy applied to women's health. J Womens Health..

[8] Ssewanyana D, Mwangala PN, Marsh V, Jao I, van Baar A, Newton CR (2018). Socio-ecological determinants of alcohol, tobacco and drug use behavior of adolescents in Kilifi County at the Kenyan coast. J Health Psychol..

[9] Oshri A, Carlson MW, Kwon JA, Zeichner A, Wickrama KK (2017). Developmental growth trajectories of self-esteem in adolescence: associations with child neglect and drug use and abuse in young adulthood. J Youth Adolesc..

[10] Kidwell RE, Eddleston KA, Kellermanns FW (2018). Learning bad habits across generations: how negative imprints affect human resource management in the family firm. Hum Resour Manag Rev..

[11] Fairman BJ, Simons-Morton B, Haynie DL, Liu D, Goldstein RB, Hingson RW (2019). State alcohol policies, taxes and availability as predictors of adolescent binge drinking trajectories into early adulthood. Addiction..

[12] Fawcett SB, Paine-Andrews A, Francisco V (1994). Preventing adolescent substance abuse: an action planning guide for community-based initiatives.

[13] Dalui A, Banerjee S, Roy RP, Ray S, Mondal R, Das DK (2018). Prevalence, Pattern and correlates of substance use among adolescents in a slum of Burdwan municipality, West Bengal: a community based study. Ind J Youth Adol Health..

[14] Fraser-Thomas JL, Côté J, Deakin J (2005). Youth sport programs: an avenue to foster positive youth development. Phys Educ Sport Pedagogy..

[15] Garfein RS, Golub ET, Greenberg AE, Hagan H, Hanson DL, Hudson SM (2007). A peer-education intervention to reduce injection risk behaviors for HIV and hepatitis C virus infection in young injection drug users. AIDS..

[16] James-Burdumy S, Goesling B, Deke J, Einspruch E (2010). The Effectiveness of Mandatory-Random Student Drug Testing, NCEE 2010-4025. Natl Cent Educ Eval Reg Assist..

[17] Dammert L, Sarmiento K (2019). Corruption, organized crime and regional governments in Peru. Corruption in Latin America.

[18] Ellis S (2009). West Africa's international drug trade. Afr Aff..

